# Development of a quantitative PCR assay for the detection and enumeration of a potentially ciguatoxin-producing dinoflagellate, *Gambierdiscus lapillus* (Gonyaulacales, Dinophyceae)

**DOI:** 10.1371/journal.pone.0224664

**Published:** 2019-11-15

**Authors:** Anna Liza Kretzschmar, Arjun Verma, Gurjeet Kohli, Shauna Murray

**Affiliations:** 1 Climate Change Cluster (C3), University of Technology Sydney, Ultimo, New South Wales, Australia; 2 ithree institute (i3), University of Technology Sydney, Ultimo, New South Wales, Australia; 3 Alfred Wegener-Institut Helmholtz-Zentrum fr Polar- und Meeresforschung, Bremerhaven, Germany; University of Helsinki, FINLAND

## Abstract

Ciguatera fish poisoning (CFP) is an illness contracted through the ingestion of seafood containing ciguatoxins. It is prevalent in tropical regions worldwide, including in Australia. Ciguatoxins are produced by some species of *Gambierdiscus*. Therefore, screening of *Gambierdiscus* species identification through quantitative PCR (qPCR), along with the determination of species toxicity, can be useful in monitoring potential ciguatera risk in these regions. In Australia, CFP is prevalent in tropical Queensland and increasingly in sub-tropical regions of Australia, but has a report rate of approximately 10%. Yet the identity, distribution and abundance of ciguatoxin producing *Gambierdiscus* spp. is largely unknown. In this study, we developed a rapid qPCR assay to quantify the presence and abundance of *Gambierdiscus lapillus*, a likely ciguatoxic species first described from Australia. We assessed the specificity and efficiency of the qPCR assay. The assay was tested on 25 environmental samples from the Heron Island reef in the southern Great Barrier Reef, a ciguatera endemic region, to determine the presence and patchiness of this species across samples from *Chnoospora* sp., *Padina* sp. and *Sargassum* sp. macroalgal hosts.

## Introduction

**Fig 1:**
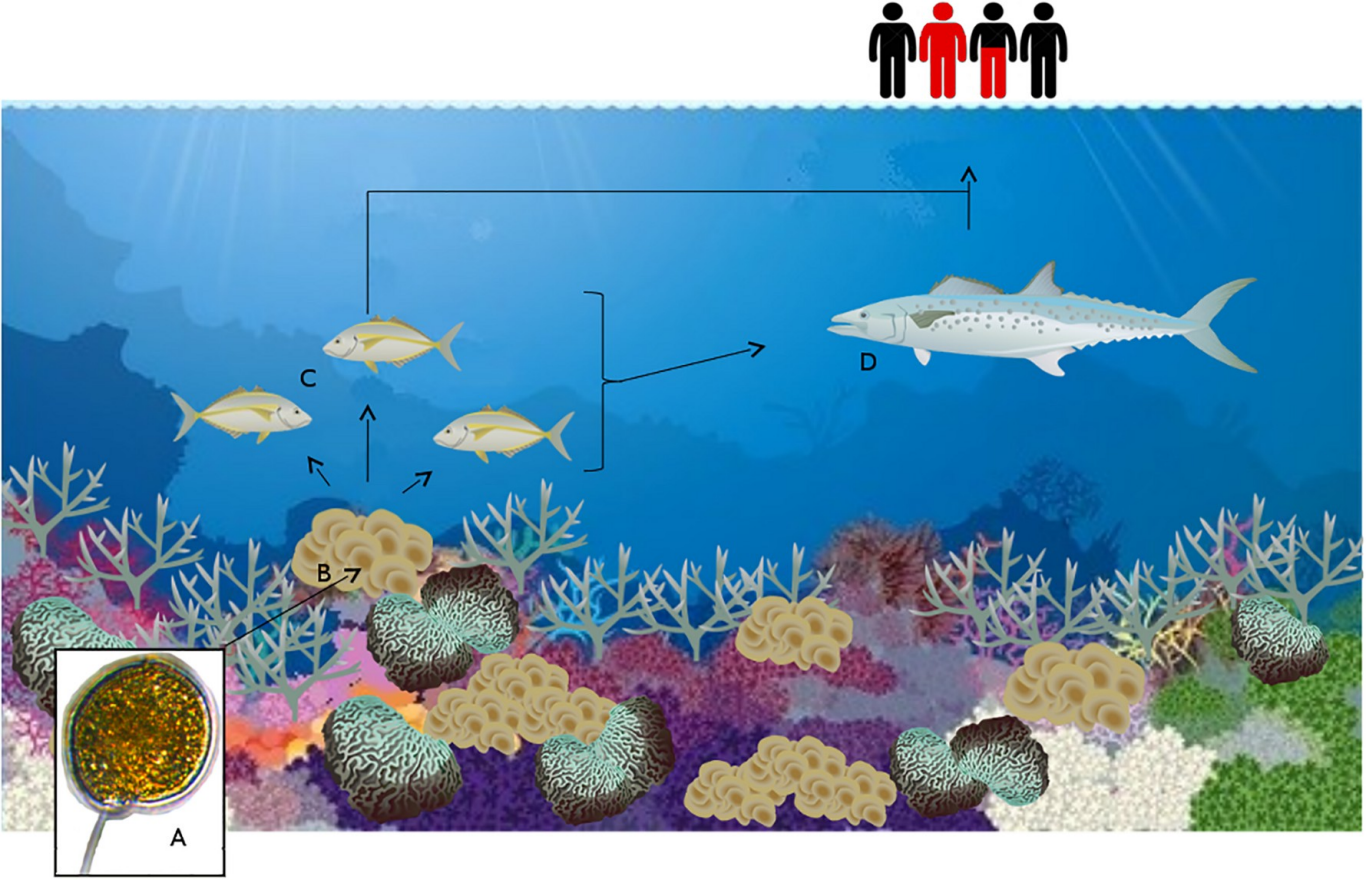
The mechanism of bioaccumulation of CTXs. *Gambierdiscus* (for example *G*. *polynesiensis* CG14 (A)) at the base of the food web inhabiting the macroalgae *Padina* spp. (B) [1]. A herbivore, here a white trevally (*Pseudocaranx dentex*) (C) [2] consumes CTX from *G*. *polynesiensis* along with the macroalgae, which then either passes directly to humans through consumption, or through an intermediary piscivorous vector such as Australian spotted mackerel (*Scomberomorus munroi*) (D) [3]. As an example, the red portion of the humans at the top of the food chain representing the 37.8% of the population in New Caledonia contracting ciguatera during their lifetime [4]. Image of *G*. *polynesiensis* (strain CG15) taken by A. L. Kretzschmar, 2016, Nikon Eclipse TS100 equipped with an Infinite Luminera 1 camera.

Benthic dinoflagellates of the genus *Gambierdiscus* Adachi & Fukuyo produce ciguatoxins (CTX), which can accumulate in humans via consumption of contaminated seafood and cause ciguatera fish poisoning (CFP) (Fig 1).

The symptoms of CFP are largely gastrointestinal and neurotoxic, however, in severe cases, further complications such as cardiovascular or severe neurological symptoms can appear [5]. In the most extreme cases, CFP can result in death [6]. Species of *Gambierdiscus* are predominantly epiphytic, growing on macroalgae and other substrates such as coral detritus. *Gambierdiscus* spp. can vary in the production of CTXs [7]. If a particular *Gambierdiscus* sp. is a CTX producer, and inhabits a palatable macroalgal substrate, the toxins can bioaccumulate in herbivorous fish with the potential to accumulate though the food chain to cause CFP in humans [8, 9, 10] (Fig 1).

*Gambierdiscus* was first described in 1977, with the type species *G*. *toxicus* [11]. The genus remained monotypic for 18 years until the discovery of a second species *G*. *belizeanus* [12]. To date, the genus comprises 18 described species and 4 ribo/species types [11, 12, 13, 14, 15, 16, 17, 18, 19, 20, 21, 22, 23, 24, 25]. Since 2014, 8 new species have been described, in part due to the increasing ease and availability of molecular genetic techniques. A major revision of the *Gambierdiscus* species taxonomy was undertaken by Litaker et al. (2009) [17]. Reports of *Gambierdiscus* spp. identified based on morphology alone, prior to this revision need to be considered with caution, as several new *Gambierdiscus* spp. were described and the previously accepted morphological features used for identification were no longer considered sufficient for distinguishing some species [26, 27, 28]. Further, even with the morphologically distinguishing features accepted today, intra-species variation and inter-species similarities can cause misidentification [21, 29, 30]. Hence, molecular genetic tools are important for determining the distribution and abundance of *Gambierdiscus* species and assessing the risk of CFP in that region [21, 29]. *Gambierdiscus* spp. produce a suite of different polyketide compounds—CTX, maitotoxin (MTX), gambierone, gambieric acid and gambierol have been characterised to date [31, 32, 33, 34, 35]. While any of these can contribute to toxicity, the toxin profile of many *Gambierdiscus* species is not well understood and only CTX has been clearly linked to CFP in humans [8, 9]. Many different assays have been used to determine CTX toxicity [36], such as the mouse bioassays and neuroblastoma cell-line bioassays, however species/strain specific toxin profiles need to be elucidated with liquid chromatography-mass spectrometry/mass spectrometry (LC-MS/MS) in order to characterise individual toxin congeners [37]. The toxin profile of *Gambierdiscus polynesiensis* is one of the only *Gambierdiscus* spp. whose production of CTX congeners (P-CTX-3B, P-CTX-3C, P-CTX-4A, P-CTX-4B and M-seco-CTX-3C) has been verified by LC-MS/MS in isolates from French Polynesia and the Cook Islands, and is thought to be the principal cause of CFP in the Pacific region [38, 39]. However recently, a *G*. *polynesiensis* strain isolated from the Kermadec Islands, in the Pacific Ocean, did not exhibit CTX toxins detectable by LC-MS/MS [40]. This demonstrated that intra-species toxin production can vary.

*Gambierdiscus lapillus* was recently described from Heron Island in the Great Barrier Reef (GBR) and it is likely part of the ciguateric web in that region [21]. Genetically, the species is closely related to *G*. *balechii*, *G*. *belizeanus*, *G*. *pacificus*, *G*. *scabrosus*, *G*. *toxicus*, *G*. sp. type 5 and *G*. ribotype 2 [18, 21]. An uncharacterised peak in the CTX phase of several strains of *G*. *lapillus* extracts was reported via LC-MS/MS, which did not match any available CTX standards (CTX-3B, CTX-3C, CTX-4A, CTX-4B) [21]. Determining the exact toxin profile of *Gambierdiscus* species requires toxin standards for comparative peak analysis, which are currently not commercially available. Bioassays provide an indication of toxin production, but not the exact congeners. Extracts from other strains of *G*. *lapillus* also show CTX-like activity in a Ca2+ influx SH-SY5Y cell Fluorescent Imaging Plate Reader bioassay [41], and their LC-MS-MS profiles show and uncharacteristic peak in the CTX phase but none of the typical CTX congeners. Therefore, this species likely produces previously uncharacterised CTX congener(s), and its production of CTX compounds requires further investigation. As CFP is endemic around the GBR, this species needs to be accurately identified and monitored *in situ*.

CFP was suggested to be a”neglected tropical disease” by expert researchers in this area, supported by the Intergovernmental Oceanographic Commissions’s (IOC) Intergovernmental Panel on Harmful Algal Blooms (IPHAB), as part of the United Nations Educational, Scientific and Cultural Organization, and a Global Ciguatera Strategy was developed [36]. One element of the IOC/IPHAB Global Ciguatera Strategy is to investigate species of the genus *Gambierdiscus*, determine which species produce CTXs through LC-MS/MS and other means, and develop efficient and reliable molecular monitoring tools for the species of interest [36]. Quantitative PCR (qPCR) was specifically mentioned in this strategy as it is a useful molecular genetic screening tool, as it can give species-specific and quantitative results from DNA extracted from environmental samples [36].

Currently there is one qPCR assay to identify the overall presence of the genera *Gambierdiscus* and/or *Fukuyoa* [42 Xsmith2017molecular]. Assays for species specific identification are available for 10 of the 18described *Gambierdiscus* spp. and 2 out of 4 undescribed *Gambierdiscus* sp. types/ribotypes (Table 1). In the development of the qPCR assays for the quantification of microalgal species, several different methods have been used to quantify species [43, 44, 45]. Using a known cell number of the target species to construct standard curves for validating qPCR assays is a common strategy, however some genes, such as rRNA genes in dinoflagellates, can have gene copy numbers that vary significantly between strains. Hence comparing an assay developed with one strain as a standard might give irregular estimates of cell numbers when used for screening environmental samples [46]. An alternative method, using a synthetic oligonucleotide specifically designed for the assay tested, allows for a standard based on the amount of copies of a gene present rather than cell numbers. This has been successfully applied to *Alexandrium tamiyavanichii* [47], a species that belongs to the sister genus to *Gambierdiscus*.

**Table 1:** Published qPCR assays for *Gambierdiscus* and *Fukoyoa* spp.

Species	Method	Reference
***Gambierdiscus* spp**.		
*G*. *australes*	TaqMan Probes and SYBR Green	[43, 44]
*G*. *belizeanus*	SYBR Green	[45]
*G*. *caribaeus*	SYBR Green	[45]
*G*. *carolinianus*	SYBR Green	[45]
*G*. *carpenteri*	SYBR Green	[45]
*G*. *jejuensis*	SYBR Green	[44]
*G*. *pacificus*	SYBR Green	[43]
*G*. *polynesiensis*	SYBR Green	[43]
*G*. *scabrosus*	TaqMan Probes	[44]
*G*. *toxicus*	SYBR Green	[43]
*Gambierdiscus* sp. ribotype 2	SYBR Green	[45]
*Gambierdiscus* sp. type 3	TaqMan Probes	[44]
***Fukuyoa* spp**.		
*Fukuyoa ruetzleri*	SYBR Green	[45]
*Fukuyoa paulensis*	SYBR Green	[42]

qPCR assays are also available for 2 of the 3 species of *Fukuyoa* (Table 1), which was split from *Gambierdiscus* as a new genus in 2015 [48 Xgomez2015fukuyoa]. *Fukoyoa* spp. are of interest for monitoring purposes as they produce MTXs, but not CTXs [21], though the involvement of MTXs in CFP has not been resolved yet [49].

In Australia, outbreaks of CFP occur annually in Queensland [50]. However, due to the complicated presentation of symptoms, the reporting rate is less than 20% [51]. Annually, there have been 7–69 reported cases between 2011 and 2015 (considering the report rate, *>* 35–345 cases, see Table 2), with 2 fatalities reported in the state [52]. Cases of CFP from Spanish mackerel (*Scomberomorus commerson*) caught in NSW have been reported since 2014 [53], with five separate outbreaks affecting a total of 24 people [54]. Farrell et al. (2017) put forward a series of recommendations to manage the emerging CFP risk in New South Wales (NSW) [54].

**Table 2:** Cases of CFP reported to health authorities in Queensland, Australia.

Year	2011	2012	2013	2014	2015
Recorded CFP cases	18	7	25	69	11
Extrapolated CFP incidences	∼90	∼35	∼125	∼345	∼55

Cases collected between 2011 and 2015, based on data collected by Queensland Health [50].

Despite the prevalence of CFP in Australia, the characterization of *Gambierdiscus* species present in Australia is incomplete. A species that produces known CTX toxins has not been identified from Australia yet. Larsson et al. (2018) have identified some candidate species, two of which show some CTX-like bioactivity, which are now characterized as *G*. *holmesii* and *G*. *lewisii* [25, 41]. Over 50% of Australia’s vast coastline (~ 66,000 km) is tropical or subtropical, and may be considered potential habitat for *Gambierdiscus* spp. [21]. Seven species of *Gambierdiscus* have been identified from the sub-tropical east Australian coastline, namely *G*. *belizeanus* [55], *G*. *carpenteri* [29, 56], *G*. *holmesii* [25, 41], *G*. *honu* (based on D8-D10 large sub-unit rRNA sequence matching to a study by Richlen et al. [57]) [20], *G*. *lapillus* [21, 41], *G*. *lewisii* [25, 41] and *G*. cf. *toxicus* [58], as well as *F*. *paulensis* [48, 55]. Using high throughput amplicon sequencing of the *cob* gene, *Gambierdiscus* was identified to the genus level in Broome, Western Australia [59], indicating that this is a coastline that should be examined further for CFP risk. qPCR primers that can be used for identification in Australia for potential monitoring purposes, have been developed for *G*. *belizeanus*, *G*. *carpenteri* and *F*. *yasumotoi* [45, 48].

The aim of this study was to develop a novel qPCR assay to exclusively amplify *G*. *lapillus*. The assay was then applied to environmental samples for the detection and enumeration of *G*. *lapillus* around Heron Island, GBR, a region in which CFP cases are regularly reported. Hence this study adds to the suite of qPCR assays available to quantify organisms that contribute to CFP.

## Methods

### Clonal strains and culturing conditions

Three strains of *G*. *lapillus* and one strain of *G*. *holmesii* were isolated from Heron Island, Australia, previously described and characterised in [21, 25]. Two strains of *G*. *polynesiensis* were isolated from Rarotonga, Cook Islands (Table 3) and their identification was performed using rRNA sequencing and phylogenetic inference, as previously described [21], and sequences have been submitted to GenBank (CG14: MH930987 for D1-D3 and MH915419 for D8-D10; CG15: MH930988 for D1-D3 and MH915420 for D8-D10). The cultures were maintained in 5x diluted F/2 media [26] at 27 °C, 60mol∙-m2 ∙-s light in 12hr light to dark cycles.

**Table 3:** List of *Gambierdiscus* clonal strains used for the qPCR assay.

Species	Collection site	Collection date	Latitude	Longitude	Strain code
*G*. *holmesii*	Heron Island, Australia	July 2014	23° 4420’ S	151° 9140’ E	HG5
*G*. *lapillus*	Heron Island, Australia	July 2014	23° 4420’ S	151° 9140’ E	HG4, HG7
*G*. *polynesiensis*	Rarotonga, Cook Islands	November 2014	21° 2486’ S	159° 7286’ W	CG14, CG15

### DNA extraction and species specific primer design

Genomic DNA was extracted from strains in Table 3 using a modified hexadecyltrimethylammonium bromide (CTAB) method [60]. The purity and concentration of the extracted DNA was measured using Nanodrop (Nanodrop2000, Thermo Scientific), and the integrity of the DNA was visualised on 1% agarose gel. A unique primer set was designed for the small-subunit ribosomal RNA (SSU rRNA) region of *G*. *lapillus* based on sequences available in the GenBank reference database (accession numbers KU558929–33). The target sequences were aligned against sequences of all other *Gambierdiscus* spp. that were available on GenBank reference database, with the MUSCLE algorithm (maximum of 8 iterations) [61] used through the Geneious software, v8.1.7 [62]. Unique sites were determined manually (Table 4, alignment is available on request). Primers were synthesised by Integrated DNA Technologies (IA, USA). The primer set was tested systematically for secondary product formation for all 3 strains of *G*. *lapillus* (Table 3) via standard PCR in 25*μ*L mixture in PCR tubes. The mixture contained 0.6 *μ*M forward and reverse primer, 1 *μ*g.*μ*L^-1^ BSA (Biolabs, Arundel, Australia), 2–20 ng DNA, 12.5 *μ*L 2xEconoTaq (Lucigen Corporation, Middleton, WI, USA) and 7.5 *μ*L PCR grade water. The PCR cycling comprised of an initial 10 min step at 94 °C, followed by 30 cycles of denaturing at 94 °C for 30 sec, annealing at 60 °C for 30 sec and extension at 72 °C for 1 min, finalised with 3 minutes of extension at 72 °C. Products were visualised on a 1% agarose gel.

**Table 4:** *G*. *lapillus* specific qPCR primer set for 138bp amplicon from the SSU rRNA designed in this study.

Primer name	Synthesis direction of primer	Sequence (5’-3’)
qGlapSSU2F	Forward	TTTTTGTCCCAGGAGGGTGA
qGlapSSU2R	Reverse	TGAGGCCAAAACTCGAAAATC

### Evaluation of primer specificity

To verify primer set specificity as listed in Table 4, DNA was extracted using CTAB buffer [63] from *G*. *australes* (CAWD149 and CG61), *G*. *belizeanus* (CCMP401), *G*. *carpenteri* (UTSMER9A3), *G*. *holmesii* and (HG5) *G*. *pacificus* (CCMP1650). *G*. *cheloniae* (CAWD232) DNA was extracted using a PowerSoil DNA isolation kit (Mo Bio Inc., CA, USA). *G*. *scabrosus* (KW070922_1) DNA was extracted using DNeasy Plant Mini Kit (Qiagen, Tokyo, Japan) as per the manufacturer’s protocol. As DNA extracts for the purpose of specificity testing were supplied by four different researchers, the extraction methods varied. However, as these served as negative controls for primer specificity only, the difference in extraction methods would not be expected to impact any of the following cell enumeration methods. For all extracted samples, the presence and integrity of genomic DNA was assessed on a 1% agarose gel. The presence of PCR inhibitors was tested for each DNA extract by amplifying SSU region as per methodology in [21]. The primer set designed for *G*. *lapillus* was tested for cross-reactivity against all other *Gambierdiscus* spp. available via PCR (BioRadT100 Thermal Cycler, CA, USA) as well as *Ostreopsis* cf. o*vata*, *O*. cf. *siamensis* and *O*. *rhodesiae* from [60]. PCR amplicons were visually confirmed on 1% agarose gel.

### Evaluation of primer sensitivity

To test the primer sensitivity, qPCR assays were run with the specifications below. Initially the amplifications were screened for a single melt curve to show binding occurred at only one site in the *G*. *lapillus* genome, then calibration curves were conducted to determine the range of detection. The qPCR reaction mixture contained 10 *μL* SYBR Select Master Mix (Thermo Fisher Scientific, Australia), 7 *μ*L MilliQ water, 0.5 *μ*M forward and reverse primers and 2–20 ng DNA template, for a final volume of 20 *μ*L. Cycling conditions consisted of 10 min at 95 °C, then 40 cycles of 95 °C for 15 seconds and 60 °C for 30 seconds, followed by a temperature gradient for melt curve construction.

Standard curves were constructed to determine the efficiency of the assay, using a synthetic gene fragment (gBlocks ^®^) approach, and also to quantify species presence, using calibration curves based on DNA extracted from known cell numbers. The calibration curves for both methods were calculated (R2, PCR efficiency and regression line slope) and graphed in R version 3.2.3 [64], using R studio version 1.0.136 [65] and the ggplot2 package [66].

#### Gene based calibration curve

For the target amplicons of *G*. *lapillus*, a DNA fragment spanning the target sequence, the reverse and forward primer sites and an extra 50bp on either end was synthesised to a total length of 238bp (gBlocks ^®^ by Integrated DNA Technologies IDT, IA, USA). The molecular weight and the amount of the synthesized gene fragment was supplied by IDT, from which the exact number of copies of the gene fragment per micoliter can be calculated [46, 47]. Lyophilized gBlocks ^®^ was re-suspended in 1x TE (Tris 10 mM, 1 mM EDTA, pH8) to a concentration of 1 ng/*μ*L. The total number of the gBlocks ^®^ gene fragment in the suspension was then calculated as 2.88x1010 for *G*. *lapillus*. The stock solution was serially diluted (10-fold) and dilutions between 103 and 108 were amplified by qPCR (on StepOnePlus^™^ System by Applied Biosystems (Thermo Fisher Scientific, Waltham, MA, USA)) in triplicate.

#### Cell based calibration curve

Two strains of *G*. *lapillus* (HG4 and HG7) were used to construct cell based standard curves. Cells were counted under a Nikon Eclipse TS100 (Australia) microscope using a Sedgwick Rafter counting chamber. DNA was extracted with the FastDNA spin kit for soil by MP Biomedicals (CA, USA), as per the manufacturer’s instructions. The gDNA extracts were 10-fold serially diluted. Dilutions ranging from 3880 to 0.04 cells and 5328 to 0.05 for HG4 and HG7 respectively. Samples were amplified via qPCR on StepOnePlus^™^ System by Applied Biosystems (Thermo Fisher Scientific, Waltham, MA, USA) in triplicates.

### Determination of gene copies per cell for *G*. *lapillus*

To determine the mean SSU rRNA copies per cell, a 10 fold dilution series for strains HG4 and HG7 were used, as described previously. These dilution series were based on DNA extracted from known numbers of cells, and then serially diluted (3880 to 0.04 cells and 5328 to 0.05 for HG4 and HG7 respectively).

The slope of the linear regression of SSU copies was used to determine copy number by correlating the qPCR detection of the gene based calibration curves and cell numbers. This slope of the linear regression was then used to determine the gene copy number per cell [47].

### Screening environmental samples for *G*. *lapillus*

**Fig 2:**
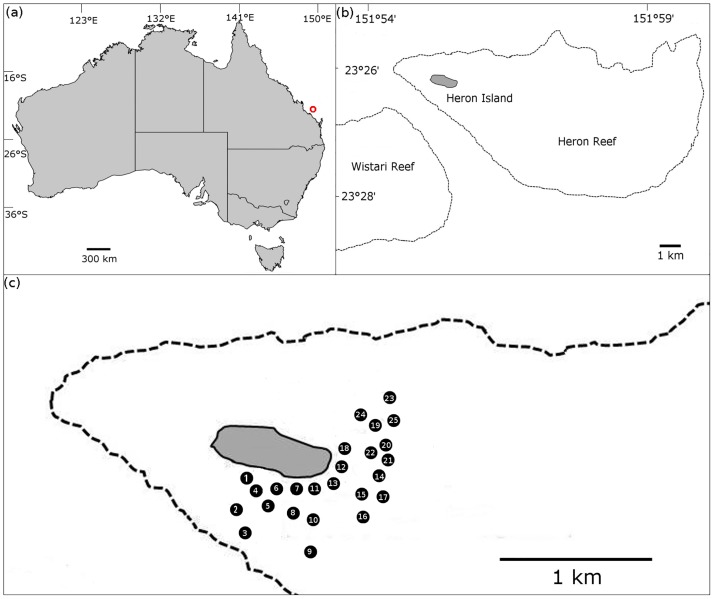
Sampling site. (A) Map of Australia, with the position of Heron Island (red circle); (B) Heron Island including surrounding reefs (dotted lines); (C) Approximate location of sampling sites around Heron Island. Map adapted from Kretzschmar et al. (2017) [21] and edited in the GNU Image Manipulation Program 2.8 (http://gimp.org).

Around Heron Reef (Fig 2) 25 sites (within 1km from the shore) were sampled in October 2015, as spatial replicates (A, B, C) within a 2m radius. Representatives of three genera of macroalgae that commonly grow on this reef, *Chnoospora* sp., *Padina* sp. and *Saragassum* sp., were sampled for the presence of epiphytic *Gambierdiscus* spp. For each sample, approximately 200 g of macroalgae was collected from approximately 1 m deep water at low tide and briefly placed in plastic bags containing 200 to 300 mL of ambient seawater. They were shaken vigorously for 5 min to detach the epiphytic dinoflagellates from the macroalgal samples. This seawater was passed through *>* 120 μm mesh filter to remove any remaining larger fauna and debris. The collected seawater was centrifuged at 500 rcf. The supernatant was discarded and the pellet was dissolved in 10 mL RNAlater (Ambion, Austin, TX, USA) for preservation and stored at 4° C. Community DNA was extracted via modified CTAB method [60]. Samples were screened in triplicate for both *G*. *lapillus* on a StepOnePlus^™^ System by Applied Biosystems (Thermo Fisher Scientific, Waltham, MA, USA). The amplification threshold was compared to the cell based calibration curve using the *G*. *lapillus* type species, HG7. The cells.g^-1^ wet weight macroalgae was calculated by determining the proportion of the total volume of DNA extract used in an individual qPCR reaction (i.e. 1 μL DNA per qPCR reaction from a 50 μL total DNA extraction volume = 0.02), quantifying the equivalent number of cells detected per qPCR reaction using the standard curve and multiplying this to determine total 200 cells.g^-1^ wet weight macroalgal sample. Normality of the data (cell numbers per macroalgal host) was tested using Shapiro test and a Bartlett test of homogeneity of variance, a Kruskal-Wallis test was performed to determine significance; in R v 3.2.3 [64] using R studio v 1.0.136 [65].

## Results

### Evaluation of primer specificity

The qGlapSSU2F—qGlapSSU2R primer pair (Table 4) amplified product of 138 bp for all five strains of *G*. *lapillus* (Table 5), with a single peak at the same temperature for each strain in the melt curve. No signal for primer dimers or unspecific amplification was detected. While genomic DNA was visible for each strain on the agarose gel (gDNA band, Table 5) and the DNA could be amplified via PCR (SSU PCR amplification, Table 5), no cross-reaction was observed for genetically closely related species *G*. *belizeanus*, *G*. *cheloniae*, *G*. *pacificus* and *G*. *scabrosus*. Other species of *Gambierdiscus* from different clades, *G*. *australes*, *G*. *carpenteri*, *G*. *holmesii* and *G*. *polynesiensis* (Table 5) were also not amplified using this primer set [13, 21].

**Table 5:** Cross-reactivity of the qPCR primer set.

Template	Strain code	gDNA gel band	SSU PCR amplification	GlapSSU2F-GlapSSU2R
*G*. *australes*	CAWD149	+	+	-
	CG61	+	+	-
*G*. *belizeanus*	CCMP401	+	+	-
*G*. *carpenteri*	UTSMER9A3	+	+	-
*G*. *cheloniae*	CAWD232	+	+	-
*G*. *holmesii*	HG5	+	+	-
*G*. *lapillus*	HG1	+	+	+
	HG4	+	+	+
	HG6	+	+	+
	HG7	+	+	+
	HG26	+	+	+
*G*. *pacificus*	CCMP1650	+	+	-
*G*. *polynesiensis*	CG14	+	+	-
	CG15	+	+	-
*G*. *scabrosus*	KW070922_1	+	+	-
*O*. cf. *ovata*	HER27	+	+	-
*O*. *rhodesiae*	HER26	+	+	-
*O*. cf. *siamensis*	HER24	+	+	-

Strains of *Gambierdiscus* and *Ostreopsis* spp. tested for qPCR primer set cross-reactivity, as well as visual confirmation of genomic DNA on agarose gel and DNA extract viability for PCR by SSU amplification.

### Evaluation of primer sensitivity

**Fig 3:**
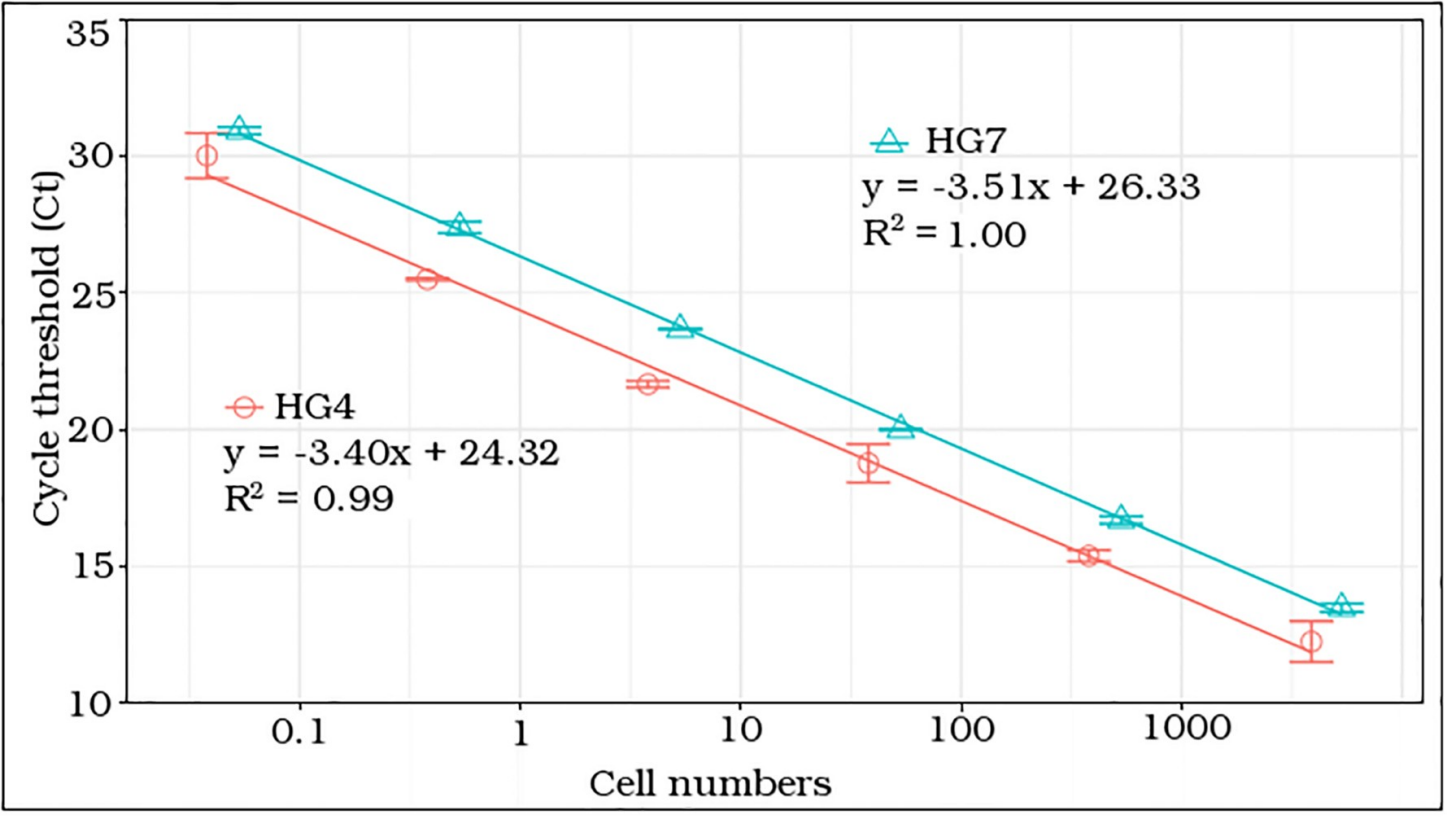
qPCR cell based standard curves of *G*. *lapillus* strains. HG4 (circle) and HG7 (triangle). Error bars represent the deviation of technical replicates during reactions; x-axis is log scale.

The cell-based standard curves for *G*. *lapillus* (HG4 and HG7, Fig 3a) showed high linearity with R2 approaching 1.00. The slope for the Ct vs. log 10 cell for HG4 was -3.4, which corresponds to an efficiency 96.8%; and -3.51, which corresponds to an efficiency of 92.7% for HG7 (Fig 3). The linear detection for both *G*. *lapillus* isolates covered five orders of magnitude. The lowest number of cells detected were 0.04 and 0.05 cells for HG4 and HG7 respectively (Fig 3a).

**Fig 4:**
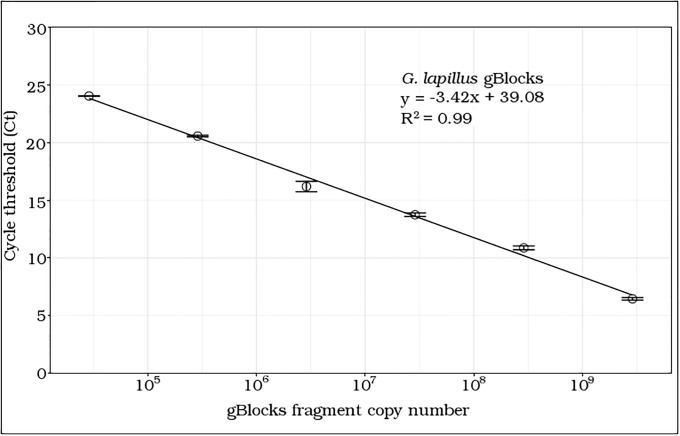
qPCR gene based standard curves of *G*. *lapillus*. Error bars represent the deviation of technical replicates during reactions; x-axis is log scale.

The gene based (gBlocks ^®^) standard curve for *G*. *lapillus* covered linear detection over five orders of magnitude, with a slope of -3.42, and a PCR efficiency of 96% (Fig 4). The detection limit tested was less than 105 gene copy numbers. The Ct for the lowest gene copy number tested was less than 25, so it is likely that the sensitivity is lower than 105 gene copy numbers (Fig 4).

### Quantification of SSU rRNA copy number per cell of *G*. *lapillus*

The detectable SSU copies for *G*. *lapillus* were 2.24 x 104 and 5.85 x 103 copies per cell for HG4 and HG7 respectively.

### Screening environmental samples for *G*. *lapillus* abundance

**Fig 5:**
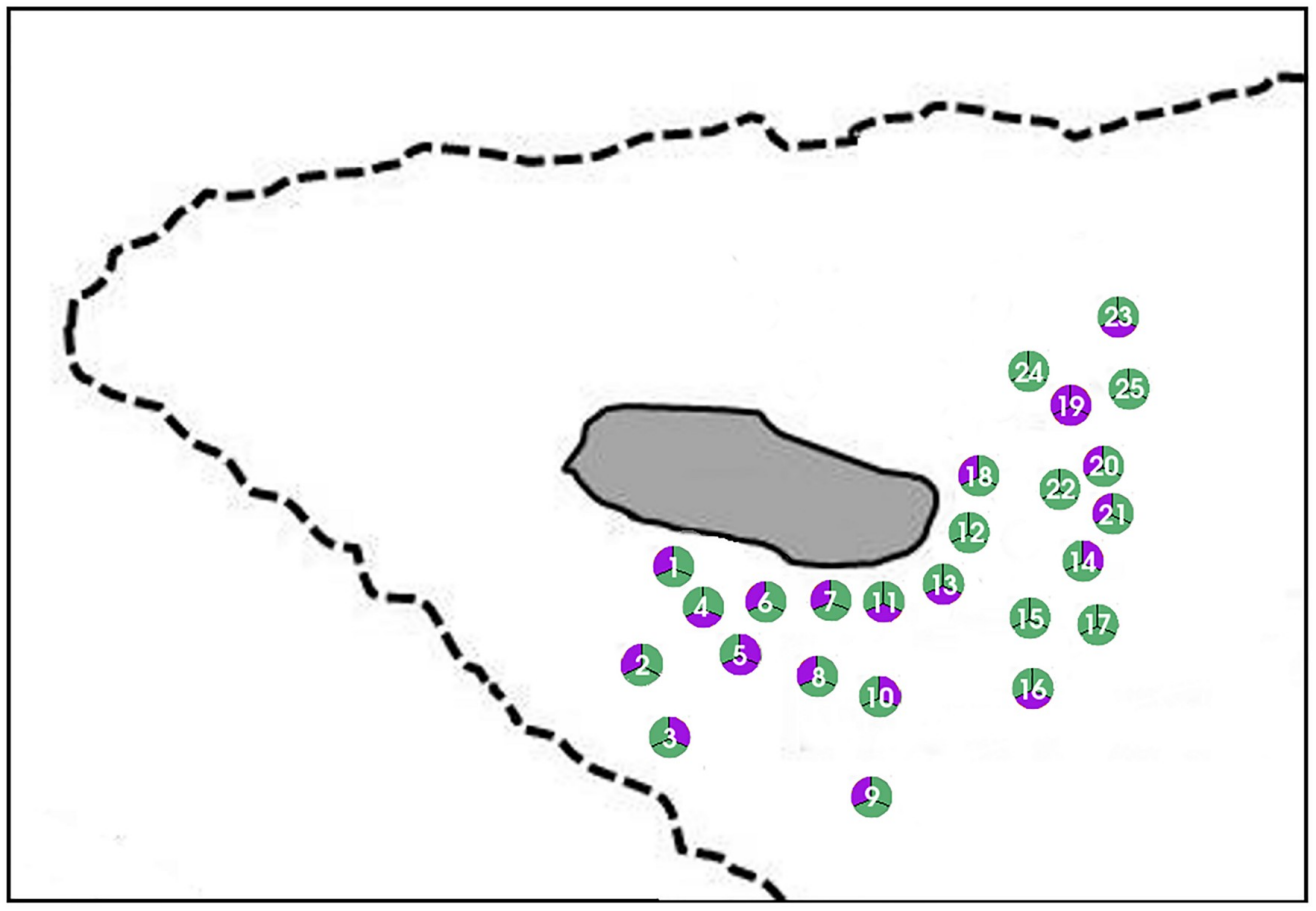
*G*. *lapillus* presence at the macroalgal sampling sites around Heron Island. The spatial replicates for each site are set up as shown in (A); the sites in (B) linked to numbering in Fig 2 where positive (green) and negative (purple) as per S1 Table.

**Fig 6:**
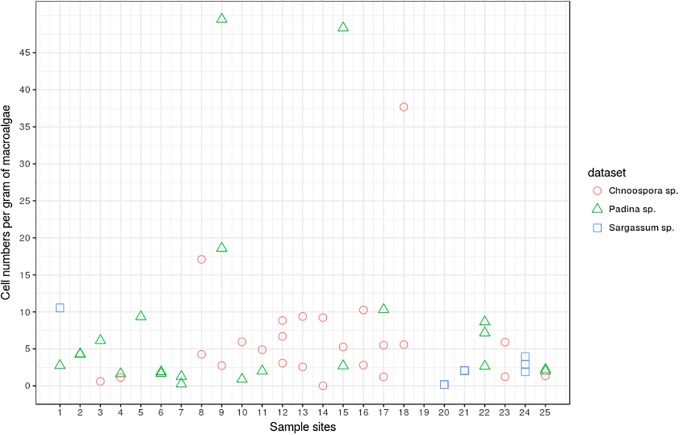
Detection of *G*. *lapillus* per spatial replicate at each macroalgal sampling site. Cell numbers were normalised to the HG7 standard curve (Fig 3A). Also shown are the spatial replicates per macroalgal substrate where *Chnoospora* sp. samples are represented by circles, *Padina* sp. by squares and *Sargassum* sp. by crosses (S1 Table).

To evaluate the adequacy of the *G*. *lapillus* qPCR assay for environmental screening, the assay was applied to environmental community DNA extracts collected from macroalgal samples around Heron Island (Fig 2). A relatively low cell abundance was detectable for *G*. *lapillus*. Ct values for *G*. *lapillus* detection in environmental samples were calibrated to the HG7 standard curve and calculated as cells.g−1 wet weight macroalgae (S1 Table). *G*. *lapillus* was detected across 24 of the 25 sampling sites. At sites at which *G*. *lapillus* was present, it showed a patchy distribution, being present at two of the three spatial replicates in the majority of samples (17 of 25 sample sites), followed by all three spatial replicates testing positive (6 out of 25 sites) and at one site only one of the spatial replicates was positive (Fig 5). *G*. *lapillus* was detected at 71 out of the 75 spatial replicates, specifically at 24/32, 22/33 and 8/10 samples from *Chnoospora* sp., *Padina* sp. and *Saragassum* sp. as substrate respectively (S1 Table). Patchiness was also found in the abundance as well as the distribution of *G*. *lapillus*, from 0.24 cells.g−1 wet weight macroalgae to 49.51 cells.g−1 wet weight macroalgae, with a mean of 5.84 cells.g−1 wet weight macroalgae. For example, (4A—*Chnoospora* sp.) and (4B—*Padina* sp.) hosted comparable cell numbers (1.12 cells and 1.65 cells.g−1 wet weight algae respectively) while no *G*. *lapillus* cells were detected on (4C—*Padina* sp.). Only at one of 25 sampling sites, no *G*. *lapillus* presence was detected across all three spatial replicates (19A, B, C). At all other sites, the presence of *G*. *lapillus* varied between spatial replicates but did not significantly differ between macroalgal host or location (chi-squared = 2.1453, *p*-value = 0.3421) (Fig 6).

## Discussion

The aim of the study was to design and validate a species-specific qPCR assay for quantification of *G*. *lapillus*, a species that may produce CTX-like toxins in the Australian GBR region. Species-specific qPCR primers with high specificity and sensitivity were developed and the SSU copy number for two strains were determined, and were found to differ from one another, as one strain had more than four times the number of genomic rRNA copies. This study also established that this primer set was effective in measuring the abundance and distribution of *G*. *lapillus* at the Heron Island reef. The cross-reactivity of primers designed in this study showed high specificity for both *G*. *lapillus* strains while not amplifying when tested against other, closely related *Gambierdiscus* species (based on target species comparison of the SSU region in Fig 2 in [21]). Standard curves were constructed for two strains of *G*. *lapillus* for which the primers showed high linearity and amplification efficiency (Fig 3). Hence, this primer set is an accurate and reproducible molecular tool to enumerate the target species exclusively from environmental community DNA extracts. Due to the putative CTX production of *G*. *lapillus* [21, 41], the presence and distribution of this species is of interest in Australia where the causative organisms for CFP are yet to be established.

As CFP risk is linked to the abundance of *Gambierdiscus* species producing CTXs [36, 67], it was important to establish a quantitative assay for detection. We validated a synthetic gene fragment standard curve of the target region (gBlocks ^®^) and compared this to cell standard curves to establish an ‘absolute’ qPCR assay [48, 68]. Further, we determined the copy SSU rRNA number for two strains of *G*. *lapillus* (HG4 and HG7). The copy number for *G*. *lapillus* (5,855.3 to 22,430.3 rRNA copies per cell) were comparable to the copy numbers determined by Vandersea et al. (2012), which ranged from 690 rRNA copies for *G*. *belizeanus* to 21,498 copies for *G*. *caribaeus* [45]. In comparison, the cell copy numbers determined by Nishimura et al. (2016) ranged from 532,000 copies for *G*. *scabrosus* and 2,261,000 for *G*. sp. type 3 [44]. While the difference in rRNA copy numbers may be due to inter-species differences, or even intra-species as per the *G*. *lapillus* results, Nishimura et al. (2016) argued that the difference could be underestimation of rRNA copy numbers due to ‘ghost’ cells (cells that look viable during cell counts under the microscope, but which are not living and therefore do not contain amplifiable DNA) [44, 68]. The difference observed in the SSU copy numbers between the two strains of *G*. *lapillus* could similarly be due to ghost cells. Further to that, variation in DNA extraction efficiency could also contribute to the difference in observed SSU copy numbers between the *G*. *lapillus* strains [48]. Alternatively, these differences in copy number may simply reflect intraspecific variation in rRNA copy numbers in dinoflagellates more broadly, which have been shown to span orders of magnitude in several species of *Alexandrium* species [69, 70]. The results presented highlight the importance of carefully verifying qPCR assays based on rRNA genes using multiple local strains as their target gene copy numbers might vary, but also the necessity and possible issues that can arise while constructing “absolute” standard curves. Tentatively, the difference of this magnitude in SSU copy numbers may lead to considerably different abundance estimates of *G*. *lapillus*. As the variation between the two strains tested is within the observed variation reported by Nishimura et al. (2016) from single cell qPCR experiments for rRNA copy number elucidation, the difference reported here is likely representative of biological intra-strain variation rather than methodological artefacts [44]. A 5-fold difference in toxicity between the same HG4 and HG7 strains for *G*. *lapillus* was also reported by Kretzschmar et al. (2017), and there was a noticeable difference in growth rate between the two strains observed (but not quantified) in this study [21]. The mounting evidence of intra-strain variability in toxicity, detectable rRNA copy numbers and potentially growth rate implies that care must be taken when interpreting qPCR based cell enumeration as a method of understanding potential CFP risk, and requires further investigation. The qPCR assay was successfully tested on environmental DNA extracts from around Heron Island, and gave some insight into *G*. *lapillus* distribution and abundance. The qPCR assay detected *G*. *lapillus* at all bar one of the sites tested (Fig 5). Within the spatial replicates, the distribution of *G*. *lapillus* was patchy, as 24 of the 25 sites included at least one replicate with no *G*. *lapillus* present (Fig 5). Patchiness in the distribution of *Gambierdiscus* species has previously been reported in a study of 7 *Bryothamnion* macroalgae spaced 5 to 10 cm apart, in which 5 to 70 cells.g^-1^ algae were found [68]. There was no significant difference in the presence/absence of *G*. *lapillus* cells observed as per the macroalgal host, *Chnoospora* sp., *Padina* sp. or *Sargassum* sp. Motile behaviour has been observed previously in the field at various time points [71, 72]. Parsons et al. (2011) reported *Gambierdiscus* sp. behaviour as facultative epiphytes during lab scale experiments, as cells showed attachment as well as motile stages over time in the presence of different macroalgae [73]. Taylor & Gustavson (1983) reported that *Gambierdiscus* cells were captured in plankton tows by de Silva in 1956 but reported as *Goniodoma* [74]. Motility could be a factor for the patchy distribution observed in the spatial replicates. Across spatial replicates where *G*. *lapillus* was detected, cell densities were consistent (Fig 6). The average cell density of *G*. *lapillus* 5.84 cells.g−1 wet weight macroalgae, which is comparable to the cell densities recorded by Nishimura et al. (2016) in their environmental screening (Table 4 in [48]).

As many authors have pointed out (e.g. [15, 36, 73, 75, 76, 77, 78]), there are several difficulties in determining precise quantification of *Gambierdiscus* species on macroalgae in order to assess potential CFP risk. Due to the difference in habitable surface area between samples taken from structurally diverse macroalgae, including those sampled in this study (*Chnoospora* sp., *Padina* sp. and *Sargassum* sp.), the potential habitable space is difficult to compare. Further, to assess CFP risk in a given area, the properties of the macroalgae with *Gambierdiscus* epiphytes need to be considered. If the macroalgae is structurally or chemically defended against herbivory, any CTX produced by the epiphytes is unlikely to enter the food chain and cause CFP [77]. Due to the difficulty in quantifying *Gambierdiscus* spp. on a particular substrate, Tester et al. (2014) proposed have the use of an artificial substrate (commonly available black fibreglass screen of a known surface area) and a standardised sampling method [76]. Molecular analysis, such as species specific qPCR, based on this standardised sampling method would be directly comparable across sampling sites and times. Adopting this approach for future monitoring studies is recommended.

## Conclusion

The qPCR assay developed in this study is an accurate molecular tool to detect and enumerate the presence of *G*. *lapillus* in environmental samples. The assay was shown to be highly sensitive and accurately detected 0.05 to over 4000 cells for *G*. *lapillus*. Although the toxin profile of *G*. *lapillus* has not been completely defined, it may produce uncharacterised CTX congeners [21, 41] and would therefore be part of the ciguateric web in Australia. The assay was applied to samples from 25 sites around Heron Island on the GBR, which found that *G*. *lapillus* was commonly present, but had a patchy spatial distribution and abundance. The development and validation of a quantitative monitoring tool presented here for *G*. *lapillus* is in line with Element 1 of the Global Ciguatera Strategy [36].

## Supporting information

S1 Table Screening of macroalgal samples for G. lapillus and cell density estimates via qPCR. Cell numbers were modeled on the type strain HG7. N/D denotes not detected.
(DOCX)
