# Supplementary material for: Development of a quantitative PCR assay for the detection and enumeration of a potentially ciguatoxin-producing dinoflagellate, Gambierdiscus lapillus (Gonyaulacales, Dinophyceae)
Source: PLoS One. 2019 Nov 15;14(11):e0224664. doi: 10.1371/journal.pone.0224664 (PMC6857910; doi:10.1371/journal.pone.0224664)
Supplement: S1 Table — Cell numbers were modeled on the type strain HG7. N/D denotes not detected. (DOCX) [file pone.0224664.s001.docx]

### Supplementary Material

Table S1: Screening of macroalgal samples for *G. lapillus* and cell density estimates via qPCR. Cell numbers were modeled on the type strain HG7. N/D denotes not detected.

| **Sample** **ID** | **Spatial** **replicate** | **Macroalgal** **substrate** | ***G. lapillus* cells per gram macroalgae** |
| --- | --- | --- | --- |
| 1 | A | *Padina* sp. | N/D |
| 1 | B | *Sargassum* sp. | 10.55 |
| 1 | C | *Padina* sp. | 2.75 |
| 2 | A | *Padina* sp. | N/D |
| 2 | B | *Padina* sp. | 4.33 |
| 2 | C | *Padina* sp. | 4.27 |
| 3 | A | *Padina* sp. | 6.13 |
| 3 | B | *Chnoospora* sp*.* | 0.62 |
| 3 | C | *Padina* sp. | N/D |
| 4 | A | *Chnoospora* sp. | 1.12 |
| 4 | B | *Padina* sp. | 1.65 |
| 4 | C | *Padina* sp. | N/D |
| 5 | A | *Padina* sp. | 9.35 |
| 5 | B | *Padina* sp. | N/D |
| 5 | C | *Padina* sp. | N/D |
| 6 | A | *Chnoospora* sp. | N/D |
| 6 | B | *Padina* sp. | 1.69 |
| 6 | C | *Padina* sp. | 1.92 |
| 7 | A | *Padina* sp. | N/D |
| 7 | B | *Padina* sp. | 0.26 |
| 7 | C | *Padina* sp. | 1.29 |
| 8 | A | *Chnoospora* sp. | N/D |
| 8 | B | *Chnoospora* sp. | 17.09 |
| 8 | C | *Chnoospora* sp. | 4.27 |
| 9 | A | *Chnoospora* sp. | N/D |
| 9 | B | *Padina* sp. | 49.51 |
| 9 | C | *Padina* sp. | 18.58 |
| 10 | A | *Padina* sp. | 0.91 |
| 10 | B | *Padina* sp. | N/D |
| 10 | C | *Chnoospora* sp. | 5.95 |
| 11 | A | *Padina* sp. | 2.01 |
| 11 | B | *Chnoospora* sp. | 4.89 |
| 11 | C | *Chnoospora* sp. | N/D |
| 12 | A | *Chnoospora* sp. | 6.70 |
| 12 | B | *Chnoospora* sp. | 8.83 |
| 12 | C | *Chnoospora* sp. | 3.08 |
| 13 | A | *Chnoospora* sp. | 2.58 |
| 13 | B | *Chnoospora* sp. | 9.39 |
| 13 | C | *Chnoospora* sp. | N/D |
| 14 | A | *Chnoospora* sp. | 0.02 |
| 14 | B | *Chnoospora* sp. | N/D |
| 14 | C | *Chnoospora* sp. | 9.24 |
| 15 | A | *Chnoospora* sp. | 5.27 |
| 15 | B | *Padina* sp. | 48.46 |
| 15 | C | *Padina* sp. | 2.71 |
| 16 | A | *Chnoospora* sp. | 2.81 |
| 16 | B | *Chnoospora* sp. | 10.26 |
| 16 | C | *Chnoospora* sp. | N/D |
| 17 | A | *Chnoospora* sp. | 5.50 |
| 17 | B | *Chnoospora* sp. | 1.23 |
| 17 | C | *Padina* sp. | 10.32 |
| 18 | A | *Chnoospora* sp. | N/D |
| 18 | B | *Chnoospora* sp. | 37.68 |
| 18 | C | *Chnoospora* sp. | 5.57 |
| 19 | A | *Padina* sp. | N/D |
| 19 | B | *Padina* sp. | N/D |
| 19 | C | *Padina* sp. | N/D |
| 20 | A | *Sargassum* sp. | N/D |
| 20 | B | *Sargassum* sp. | 0.19 |
| 20 | C | *Sargassum* sp. | 0.18 |
| 21 | A | *Sargassum* sp. | N/D |
| 21 | B | *Sargassum* sp. | 2.11 |
| 21 | C | *Sargassum* sp. | 2.05 |
| 22 | A | *Padina* sp. | 7.17 |
| 22 | B | *Padina* sp. | 2.67 |
| 22 | C | *Padina* sp. | 8.64 |
| 23 | A | *Chnoospora* sp. | 1.24 |
| 23 | B | *Chnoospora* sp. | 5.90 |
| 23 | C | *Chnoospora* sp. | N/D |
| 24 | A | *Sargassum* sp. | 1.91 |
| 24 | B | *Sargassum* sp. | 2.90 |
| 24 | C | *Sargassum* sp. | 3.97 |
| 25 | A | *Padina* sp. | 2.24 |
| 25 | B | *Chnoospora* sp. | 1.36 |
| 25 | C | *Padina* sp. | 2.00 |
